# Evaluation of Effective Dashboards: Key Concepts and Criteria

**DOI:** 10.2174/1874431101711010052

**Published:** 2017-10-24

**Authors:** Mahtab Karami, Mostafa Langarizadeh, Mansoor Fatehi

**Affiliations:** 1 Health Information Management Research Center (HIMRC), Kashan University of Medical Sciences, Isfahan Province Kashan, Iran.; 2 Iran University of Medical Sciences, Tehran Province, Tehran, Iran; 3 Medical Imaging Informatics Research and Education Center, Tehran, Iran

**Keywords:** Dashboard, Visualization, Criteria, Healthcare, Concepts, Evaluation

## Abstract

**Objective:**

The aim of this study is to offer appropriate criteria to evaluate effective dashboards for healthcare organizations.

**Method:**

In a Delphi study, a team of information technology consultants were asked to determine a set of user interface requirements for evaluating, building or selecting the dashboard. In the first round, a list of main features or criteria to be used was determined based on the panel’s rating,.

**Results:**

In this study, it was revealed that a set of key criteria for evaluating the dashboards can be used for all types of dashboards. These criteria were classified into 7 main categories including user customization, knowledge discovery, security, information delivery, alerting, visual design, and integration and system connectivity.

**Conclusion:**

Choosing good criteria for selecting effective dashboards for healthcare organizations is very critical because these organizations follow a data-intensive and technology-driven environment. This study revealed the importance of key criteria which can guarantee development of an evaluation checklist.

## 1 INTRODUCTION

A dashboard is a visualizing tool which provides awareness, trending, and both planning and actual comparisons, frequently visualized in a slick simplified user interface. With so many dashboards available, choosing an appropriate one often boils down to the users of dashboard tools [1].

This intellectual and visual tool has been used in healthcare fields, for example, 1) in the operation roomsto allocate resources and cost management, [2] in radiology departments to improve dose management and X-ray usage, [3] in emergency departments to decrease patient's length of stay and for improving capacity and workflow management, [4, 5] and in SICU to increase compatibility with ventilator bundle measures, and for decreasing rates of VPA [6].

The dashboard should have goals set by users and consistently meet their expectations. End user experience is one of the major features of dashboard software [7]. End users are information consumers who make decisions and drive change management strategies based on presented information [8].

In this regard, it is necessary for the dashboard to communicate information about the pros and cons of decision alternatives quickly, highlight factors that merit consideration, and provide information in a non-linear format to facilitate its incorporation in decision making deliberations [9].

While dashboards constantly evolve, developing evaluation criteria based on clinical relevance, efficiency, and usability is important [1]. The main aim of this study is to determine evaluation criteria of dashboards in the healthcare organizations. The results obtained from this study can help users to determine a set of user interface requirements to inform the building or selecting an appropriate dashboard for operational use.

## 2 METHODS

In this Delphi study, a review of literature was firstly performed to gain a good understanding of the criteria that contributed to evaluation of the dashboard tools. Based on the literature review, a number of features and their relevant elements were extracted to assist and inform user interface requirements. These features were known as category and the elements were known as criteria. It was then set which criterion was relevant for each category.

Next, a panel of information technology consultants composed of experts in health information management, medical informatics, and software engineering along with radiologists as end users (because a prototype of the dashboard was used in the radiology department.) were asked to confirm and rank these criteria with an open ended question included to seek further potential criteria. In this process, the panel was asked to declare their opinions about whether a criterion is necessary or not. They also were asked to rank every criterion based on its priority and the degree of importance in terms of category. In this regard, an electronic checklist including items such as criteria, yes, no, rank, and your comments were designed. In the first round, a list of the criteria to be used was determined. SPSS (ver. 16) was then employed for descriptive statistical analyses.

## 3 RESULTS

Out of the 46 experts, 42 completed their task. A total of 56 criteria statements were confirmed in the first round. Since in the first round no offers were gained, the Delphi study was completed with only one round. 56 important criteria for evaluating or building or selecting an effective dashboard were categorized into 7 groups. These are presented in Tables (**1**-**7**).

The results showed that the first category “user customization” and its four main subgroups were highly rated, except for “discussion forum” which was ranked at the level of 5. The “knowledge discovery” category covered 5 criteria with the same degree of importance. All the criteria ranked as 1. In the category of “security”, 7 criteria were of which only the “version control” criterion gained the lowest rank as 5.

There were 8 criteria included in the “information delivery” of which the “Sorting the report” was found at the lowest rank of 6. The “visual design” entailed 10 criteria of which the “single screen with no scrolling” gained the lowest ranking of 6. The “alert” category contained 10 criteria in which the “delivering alert through pager” obtained the lowest priority of 3. The “system connectivity and integration” category consisted 6 criteria of which the “integrating with portal” was considered as the lowest rank of 3.

## 4 DISCUSSION

Based on the results, the dashboard evaluation criteria were grouped into 7 major categories. The first category included “user customization”. This criterion can empower the users to response rapid changes in environment and turn their departments to agile department [10]. In this category, “discussion forum” gained the lowest priority. The discussion forum is “an application that allows a thread of communication among several users”. Low priority does not meant that this criterion is not important because this feature can enhance the collaboration between users and ultimately make better decisions, especially in times of crisis. In fact, collaboration converts the role of the dashboard from passive information interface to an active management console [7, 11].

The second category was knowledge discoveryin which all the criteria obtained high priority, because they allow users to conduct root analysis, especially when diving to discover cause of incidents or problems [11, 12].

Among the security criteria, “version control” found the lowest priority. The definition of version control is a “repository of files, often source code files of computer programs, with monitored access. All changes applied on source files are tracked along with who made such changes, why they did it, and references to problems fixed, or enhancements introduced” [13]. Therefore, it is an important feature.

Although, dashboard is not a reporting tool, however, its main use is reporting. Therefore, the way of “information delivery” is very important for dashboard design. The dashboard should meet the objectives that are defined and understood by the users on an ongoing basis. And also, the context of the contents being displayed in the dashboard should be in clarity [12, 14].

The options such as sorting, filtering, exporting, inserting, deleting, scheduling, and updating are the user’s interactions with the dashboard. Pleasurable and respectful user interaction can enhance user’s quality of work-life because it enables the users to access to information at the right time with the least amount of effort and also keeps the dashboard stable dynamically [14-16].

The sixth group was “visual design”. The dashboard should be visually appealing and engaging without overwhelming the users but make them feel comfortable and enable them to change the information layout [14]. It is necessary to adopt a concise and minimalist design in order to avoid overloading the user with information, components, contents, and navigation steps that are unnecessary. This is lead to avoid from fragmenting the information by having to scroll. The capability of “Single screen with no scrolling” caused to display useful information without screen scrolling and users can overview them quickly [14-19].

The criterion “metadata and help” informs the user about the dashboard and instructions for its use. This information should be visible or easily retrievable whenever appropriate. In this category, visual intelligence means the capability of software to provide better insight through intelligently highlighting relevant areas and values on the dashboard in response to a user's cursor movement. Intelligent presentation improves the user's ability to extract information from data” [14].

“Alerting” was the seventh category. The alerts are a mechanism to turn the focus to the exceptions, outliers and data highlights. Whether embedded in the dashboard or presented separately, alerts can be used as extra layers of abstraction to make a dashboard more useful [20]. The dashboard should be able to manage events to evaluate the performance of department against predefined goals using indicators [11, 12]. It is better to present the alerts by color coding to show levels of threats. These alerts are defined based on performance target thresholds which are derived from the yearly goals and objectives. Thresholds are defined as a target zone in green, a warning zone in yellow, and a trouble zone in red [21].

Also, agents can be used in data models for registering and managing alerts if alerts are to be shown in groups or individual [21]. Agent is a computing entity which is located in dynamic and complex environment and can autonomically sense the environment and act accordingly to complete its tasks or goals [22]. In ranking, the criterion “deliver alert through pager” found the lowest rank. It should be noted that in the design of alerts, unnecessary alarm must be avoided [3].

Finally, the seventh category was “system connectivity and integration”. The dashboard software must be coordinated with the system infrastructure in organization. It must support different versions of all operating systems used within the organization and also must fall into the domain of organization’s application servers. On the other side, the dashboard should be able to capture live data from various data sources, and if data values in the specific data source have changed, those changes should be reflected in the dashboard. It should be able to interact with standard databases such as relational or multidimensional data bases [7]. Integrating with portal obtained the lowest rank while this is a main feature to achieve the virtual dashboard [23].

## CONCLUSION

Since healthcare is a data-intensive and technology-driven environment, choosing good criteria to select effective dashboards for using in such environment is critical. This study tried to reveale the importance of key criteria which could inform the development of a checklist for evaluating the dashboards. Align with progression in dashboard technology, comparative study and user feedback will lead to further improvement in this field.

## Figures and Tables

**Table 1:** Feature of user customization and related criteria.

**Key Criteria**		**Ranking**		**Yes**	**No**
		Rank	No. (%)	No. (%)	No. (%)
Customizing Definitions	Goals	1	33 (78.6%)	42 (100%)	0
	Objectives	2	19 (45.2%)	42 (100%)	0
	Metrics	4	15 (35.7%)	42 (100%)	0
	End targets	1	10 (23.8%)	41 (97.6%)	1 (2.4%)
	calculations	3	12 (28.6%)	40 (97.5%)	2 (4.8%)
	Correlation among metrics	1	16 (38.1%)	42 (100%)	0
Categorization	Restricted access to specific metrics by different users	1	17 (40.5%)	40 (95.2%)	2 (4.8%)
	Assigning a group of users to a group of dashboards	1	10 (23.8%)	37(88.1%)	5 (11.9%)
Feedback	Attach comments to metrics	1	15 (35.7%)	35(83.3%)	7 (16.7%)
	Discussion forum among users	5	22 (52.4%)	33 (78.6%)	9 (21.4%)

**Table 2:** Feature of knowledge discovery and related criteria.

**Key Criteria**	**Ranking**		**Yes**	**No**
	Rank	No. (%)	No. (%)	No. (%)
Drill-down features	1	18 (42.9%)	42 (100%)	0
Dimensional modeling with hierarchies and levels	1	23 (54.8%)	38 (90.5%)	4 (9.5%)
Dependency analysis	1	14 (33.3%)	41 (97.6%)	1 (2.4%)
What-if analysis	1	15 (35.7%)	42 (100%)	0
Move from monitoring layer to analysis layer	1	14 (33.3%)	41 (97.6%)	1 (2.4%)

**Table 3:** Feature of security and related criteria.

**Key Criteria**	**Ranking**		**Yes**	**No**
	Rank	No. (%)	No. (%)	No. (%)
Appropriate authentication and authorization methods	1	28 (66.7%)	39 (92.9%)	3 (7.1%)
Backup and restore procedures	1	15 (35.7%)	42 (100%)	0
version control dashboards	5	10 (23.8%)	39 (92.9%)	3 (7.1%)
Audit trails	1	16 (38.1%)	41 (97.6%)	1 (2.4%)
Protecting data from change	1	16 (38.1%)	42 (100%)	0
Defining role-based security	1	17 (40.5%)	41 (97.6%)	1 (2.4%)
Automatic accessibility change by change in user roles or groups	1	20 (47.6%)	36 (85.7%)	4 (14.3%)

**Table 4:** Feature of information delivery and related criteria.

**Key Criteria**	**Ranking**		**Yes**	**No**
	Rank	No. (%)	No. (%)	No. (%)
Reasonable response time and latency	1	26 (61.9%)	42 (100%)	0
Customized layout of metrics for print	2	13 (31%)	40 (95.2%)	2 (4.8%)
Exporting information to spreadsheets, presentation slides, word, PDF, etc	1	18 (42.9%)	42 (100%)	0
Data filtering for selected reports	1	16 (38.1%)	42 (100%)	0
Sorting the report	3	10 (23.8%)	42 (100%)	0
Inserting/deleting columns	1	11 (26.2%)	40 (95.2%)	2 (4.8%)
Scheduling automatic reports	1	13 (31%)	42 (100%)	0
Updating the reports	1	12 (28.6%)	41 (97.6%)	1 (2.4%)

**Table 5:** Feature of visual design and related criteria.

**Key Criteria**	**Ranking**		**Yes**	**No**
	Rank	No. (%)	No. (%)	No. (%)
Visual intelligence to highlight areas and values	1	21 (50%)	37(88.1%)	5 (11.9%)
Table and chart on same screen	1	16 (38.1%)	37(88.1%)	5 (11.9%)
Toggling between tabular and chart views	2	13 (31%)	40 (95.2%)	2 (4.8%)
Resizing, maximize/minimize, re-ordering of zones	2	11 (26.2%)	40 (95.2%)	2 (4.8%)
Allowing different layouts	2	8 (19%)	35(83.3%)	7 (16.7%)
Inclusion of metric definition and calculation	1	18 (42.9%)	38 (90.5%)	4 (9.5%)
Linking objectives with metrics	1	15 (35.7%)	42 (100%)	0
Linking metrics together	1	13 (31%)	42 (100%)	0
Having Metadata and help	1	15 (35.7%)	39 (92.9%)	3 (7.1%)
Single screen with no scrolling	3	29 (69%)	32(76.2%)	10(23.8%)

**Table 6:** Feature of alerting and related criteria.

**Key Criteria**		**Ranking**		**Yes**	**No**
		Rank	No. (%)	No. (%)	No. (%)
Customizing and managing the alerts	Defining the alerts	1	33 (78.6%)	42 (100%)	0
	Highlighting by color coding for unexpected values	2	20 (47.6%)	38 (90.5%)	4 (9.5%)
	Determining the timing of alerts	1	15 (35.7%)	39 (92.9%)	3 (7.1%)
	Placing the alerts in context	1	16 (38.1%)	42 (100%)	0
Delivering alerts through	Dashboard website	1	15 (35.7%)	41 (97.6%)	1 (2.4%)
	Email	1	15 (35.7%)	39 (92.9%)	3 (7.1%)
	pager	3	13 (31%)	42 (100%)	0
	Cell phone	1	18 (42.9%)	33 (78.6%)	9 (21.4%)
Showing the next step to do		1	11 (26.2%)	40 (95.2%)	2 (4.8%)
Explaining the problem using text		1	20 (47.6%)	41 (97.6%)	1 (2.4%)

**Table 7:** Feature of system connectivity & integration and related criteria.

**Key Criteria**	**Ranking**		**Yes**	**No**
	Rank	No. (%)	No. (%)	No. (%)
Connectivity to a variety of data sources like OLAP cubes, Databases, Lists and Spreadsheets	1	40 (95.2%)	42 (100%)	0
Supporting different operating systems	1	18 (42.9%)	38 (90.5%)	4 (9.5%)
Integrating with portals	3	12 (28.6%)	42 (100%)	0
Integrating with other applications	1	17 (40.5)	42 (100%)	0
Recovering from software or hardware crash	2	14 (33.3%)	40 (95.2%)	2 (4.8%)
Integrating with programmatic APIs for data & metadata	2	15 (35.7%)	34 (81%)	2 (19%)
